# Exploring the Factors That Influence the Intention to Co-create Open Educational Resources: A Social Exchange Theory Perspective

**DOI:** 10.3389/fpsyg.2022.918656

**Published:** 2022-06-24

**Authors:** Xiaochen Wang, Ruisha Han, Harrison Hao Yang

**Affiliations:** ^1^College of Teacher Education, Capital Normal University, Beijing, China; ^2^College of Education, Capital Normal University, Beijing, China; ^3^Faculty of Artificial Intelligence in Education, Central China Normal University, Wuhan, China; ^4^School of Education, State University of New York at Oswego, Oswego, NY, United States

**Keywords:** open educational resources, knowledge co-creation, social exchange theory, behavioral intention, benefit, cost

## Abstract

**Purpose:**

Based on social exchange theory, this study aimed to investigate, from the cost-benefits perspective, the intention to co-create open educational resources (OER).

**Design/Methodology/Approach:**

Participants in the study included 311 undergraduate students selected from those enrolled in a course on the China University MOOC platform. Regression analysis was conducted to examine cost and benefits factors that influenced participants’ intentions to co-create OER.

**Findings:**

(1) From the perspective of benefits, expected reciprocity, increase in knowledge self-efficacy, and creative self-efficacy were found to significantly and positively impact the intention to co-create OER, while increase in internet self-efficacy was not. (2) From the perspective of cost, perceived effort and perceived privacy were found to significantly and negatively impact the intention to co-create OER, while perceived intellectual property risks were not significant.

**Originality/Value:**

Three strategies are recommended to promote the intention to co-create OER based on the findings of this study: (1) focusing on OER communities and developing reciprocity norms; (2) popularizing and promoting knowledge and use of Creative Commons copyright licenses; (3) providing easy-to-use online resource editing tools for use with OER repositories.

**Research Limitations/Implications:**

Future research should explore other ages, cultural backgrounds, and types of online learning experience to help broaden the universality of the results.

## Introduction

Open Educational Resources (OER) are a strategic opportunity to improve the quality of learning and knowledge sharing, with the ultimate goal of creating societies with inclusive access to knowledge (UNESCO, 2020; Wang et al., 2021). The sharing, exchange, and co-creation of knowledge are value-laden processes (Liu, 2008) through which knowledge is created to meet its utmost usefulness. Co-creation of knowledge is getting more attention gradually. (Zwass, 2010). Wiley’s 5R’s OER model (Retain, Reuse, Revise, Remix and Redistribute) (Wiley, 2014), is based on the idea that the value of OER lies not only in cost savings and easy access, but also in participation and co-creation in contents and materials (Nurhas et al., 2018).

In general, OER co-creation is a process in which users and instructors work together to create new OER materials. Kangas (2010) suggests that OER co-creation is not only as a social process in which new OER emerges, but also a process in which new OER is socially optimized and validated through the interaction of multiple stakeholders. Many mainstream OER repositories such as the OER Commons and OpenLearn provide online authoring and other community tools to support co-creation. However, co-creation of materials among students and educators is as yet in a nascent phase (Arinto et al., 2017), and global distribution of co-created digital materials is still low (Nurhas et al., 2018). A prior study found that users had little interest in the practice of co-creation (Rodríguez et al., 2018).

Some studies have tried to stimulate the co-creation of OER. Baldiris et al. (2017) proposed the OER Co-Evaluation model as a way to support the co-creation of inclusive and accessible OER. Rodríguez et al. (2018) discussed the sociocultural, educational, and technosocial factors that impact the co-creation of OER. Nurhas et al. (2018) identified essential social-personal and technical-environmental barriers to the co-creation of OER. Understanding what influences participants and drives their behaviors is seen as vital (Roberts et al., 2017) if one is to understand and influence co-creation and similar activities. So far, the existing literature has mostly focused on analyses from the perspectives of external support and the obstacles to co-creation. However, few studies have investigated the co-creation of OER from the perspective of individual engagement.

Based on the above considerations, this study explores the factors that influence the co-creation of OER from the perspective of social exchange theory, which address both individual and reciprocal approaches to study social behavior in the interactions of two or more parties by implementing a cost-benefit analysis. Because it is difficult to measure actual co-creation behavior, this study instead analyzed behavioral intention, defined as the willingness to try to perform a behavior, as a way to predict future co-creation behavior (Ajzen, 1991). The core issues of this paper are thus as follows:

Q1: How to understand the potential factors affecting the intention to co-create OER from a social exchange theory perspective?

Q2: What is the relationship between the intention to co-create OER and its influencing factors?

## Conceptual Framework

### Social Exchange Theory

The core theoretical assumption of social exchange theory (SET) is that all social life can be investigated as an exchange of tangible and intangible rewards and resources between/among actors (Homans, 1974) on the grounds that “all relationships have ‘give and take”’ (Kaynak and Marandu, 2006). SET has been widely adopted as one of the most influential theories used to explain social interaction information systems (Stafford, 2008), and has proven valuable in the analysis of knowledge sharing (Kankanhalli et al., 2005) and innovative user behavior in online communities (Hemetsberger, 2002). Cost-benefit analysis under SET has been used by many studies. For example, Shiau and Luo (2012) explored the factors affecting online group buying intention and satisfaction. Yan et al. (2016) analyzed knowledge sharing in online health communities. Wayne et al. (1997) brought perceived organizational support and leader-member exchange together in an integrated model of social exchange. Gould-Williams and Davies (2005) predicted the effects of human resource management practice on employee outcomes. Slack et al. (2015) explored the factors affecting employee engagement. These studies show that different contexts involve different factors of cost and benefits. So far, however, there has been little quantitative analysis of the factors that affect the intention to co-create OER from a cost-benefit perspective. It is hoped that this research will contribute to a further understanding of the factors influencing the intention to co-create OER. SET is here adopted as a theoretical framework on the grounds that cost-benefit evaluation may adequately reflect the characteristics of knowledge co-creation from the perspective of individual engagement (Vivek et al., 2012).

### Benefit Determinants of the Intention to Co-create Open Educational Resources

Social exchange theory suggests that individuals have expectations of private benefits for their contributions (Beltagui et al., 2019). These expected benefits act as motivators of human behavior that can be extrinsic or intrinsic in nature (Kankanhalli et al., 2005; Shalley et al., 2009). Extrinsic benefits include expectations of both economic and non-monetary reciprocity, (Bock et al., 2005; Fey and Furu, 2008) which can develop strong ties within a community (Roberts et al., 2014) and effectively encourage knowledge sharing as well (Wasko and Faraj, 2000; Bock et al., 2005). Few studies have recommended financial rewards for knowledge sharing (Bartol and Srivastava, 2002). Because OER are generally provided for free, economic rewards cannot be discussed as a major factor. As the main resource and value of OER, knowledge is multiplied by giving it away freely to others and thus fosters contributive behavior (Hemetsberger, 2002).

There are few empirical studies that analyze the relationship between reciprocity and knowledge co-creation. However, many studies have been performed on reciprocity and knowledge sharing, and these can potentially serve as a meaningful reference. Most studies have found a positive relationship between the two (Cabrera and Cabrera, 2005; Lin et al., 2009), while a few have found non-significant (Hung et al., 2011) or negative results (Wasko and Faraj, 2005; Chen and Hung, 2010). One possible reason for this discrepancy is that reciprocity is a double-edged sword (Preece, 2001), not only concerned with contributing, but also with receiving from the other. The contributors may feel disappointed and reduce or stop contributing when they do not get what they expect (Chandola et al., 2007).

SET defines intrinsic benefit as inherent satisfaction in a task rather than tangible or intangible rewards (Sedighi et al., 2016). In this regard, reputation (Nambisan and Baron, 2010) and happiness (Lawler and Thye, 1999) are two important factors in general social exchange. Decentralized crowdsourcing of OER is not conducive to directly establishing authority or reputation. However, self-efficacy, which is the confidence that people have that they can achieve a particular goal (Bandura, 1997), is the most important factor that can enhance participants’ self-worth, especially in an online context (Liao et al., 2013). When participants contribute knowledge and experience, their self-efficacy will be enhanced (Cabrera and Cabrera, 2002). In turn, the increase in self-efficacy has also been found to be significant in motivating the individual to engage in an Internet-based co-creation endeavor (Füller et al., 2009; Judith and Bull, 2016).

There are different types of self-efficacy and these types may fulfill different roles. Among these, knowledge self-efficacy (Kankanhalli et al., 2005; Yilmaz, 2016), Internet self-efficacy (Tamjidyamcholo et al., 2013), and creative self-efficacy (Tierney and Farmer, 2002) should be explored in the context of co-creation of OER. Researchers have reported a significant positive relationship between self-efficacy and knowledge sharing (Cabrera et al., 2006; Papadopoulos et al., 2013), self-efficacy and online community participation (Shea and Bidjerano, 2010), and self-efficacy and co-production of public services (Bovaird et al., 2015). These explorations and conclusions are a valuable basis for the current work.

### Cost Determinants of the Intention to Co-create Open Educational Resources

According to SET, costs are defined as negative outcomes from exchange behavior, which thus reduce the frequency of the behavior (Yan et al., 2016). Studies in the enterprise social media domain have found time and effort to be the most significant barriers to participation in knowledge sharing (Vuori and Okkonen, 2012). Perceived effort is defined as consisting of psychological costs and can be used to analyze participants’ decisions about sharing their knowledge with others (Sedighi et al., 2016). Prior studies have reported a significant negative relationship between perceived effort and knowledge sharing (He and Wei, 2009), perceived effort and new technology adoption (Mac Callum and Jeffrey, 2014), and perceived effort and online learning engagement (Dixson et al., 2017).

With regard to the co-creation of OER, the perceived effort mainly consists of online knowledge codification effort (Beck et al., 2015), which is defined as the amount of energy invested in the knowledge-contribution (Markus, 2001), both in terms of the time and the exertion required to codify and input information (Kankanhalli et al., 2005; Lin C. P., 2007; He and Wei, 2009). For example, specific network orchestrating capabilities are required (Möller, 2006) to re-mix and distribute knowledge created in the network (Laudien and Daxboeck, 2016). Even after contributing knowledge, there may be additional requests for clarification and assistance from knowledge recipients, which take up even more codification time from knowledge contributors (Goodman and Darr, 1998). The time and energy required for codifying knowledge can be thought of as an opportunity cost that hinders participants’ creation and editing of resources (Kankanhalli et al., 2005).

Another important cost of the co-creation of OER is perceived risk, which is always related to negative outcomes and uncertainty (Kim et al., 2015). Within the SET framework, the higher the perceived risk, the higher one perceives the potential costs to be, and hence the lower the expected net benefit and engagement to be gained (Pavlou and Gefen, 2004). Co-creation of OER tends to be open-ended and thus involves both flexibility and risk, which can dissuade participants from becoming co-creators of value (Seppä and Tanev, 2011). When it comes to online-related activities, perceived privacy risks refer to one’s subjective belief regarding the expectation of losses associated with the release of personal information to others in electronic communities (Malhotra et al., 2004; Dinev and Hart, 2006). Furthermore, different people have different understandings of intellectual property law as it relates to OER (Atkins et al., 2007). This can make participants feel extra anxiety when co-creation activities cross national borders, and this may inhibit the production and remix of OER (Joyce, 2007).

### Research Model and Hypotheses

**FIGURE 1:**
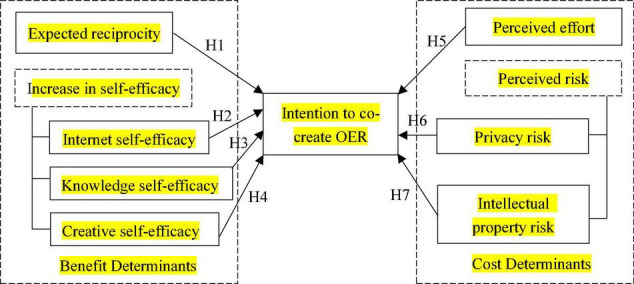
Research hypotheses of this study.

Based on the prior work reviewed above, as show in Figure 1, we propose the following hypotheses:

H1: Expected reciprocity is positively related to the intention to co-create OER;
H2: Increase in Internet self-efficacy is positively related to the intention to co-create OER;
H3: Increase in knowledge self-efficacy is positively related to the intention to co-create OER;
H4: Increase in creative self-efficacy is positively related to the intention to co-create OER;
H5: Perceived effort is negatively related to the intention to co-create OER;
H6: Perceived privacy risk is negatively related to the intention to co-create OER;
H7: Perceived intellectual property risk is negatively related to the intention to co-create OER.

## Methodology

### Participants

In total, 331 undergraduate students were selected from the course “Modern Educational Technology and Practice” in the Fall semester of the 2019–2020 academic year on the China University MOOC platform. China University MOOC platform is the largest OER repository in Chinese higher education. The course is a compulsory course for students who are in various teacher preparation programs, except those who are majoring in educational technology. It covers instructional design, ICT in education, teaching strategies, evaluation, and presentation software. Similar to most MOCC courses, students in this course are expected to engage in self-directed learning activities such as reading and reviewing digital learning resources, participating in online discussions, and completing individual and group learning tasks (Wei et al., 2022). Among the 331 samples (male = 135, female = 196), there were 58 freshmen, 124 sophomores, 96 juniors, and 53 seniors, and 68.7% of participants had contributed knowledge online before.

### Measures

In this study, the items used to operationalize the constructs were mainly adapted from previous studies and modified for use in the OER co-creation context. All constructs were measured using multiple items. All items were measured using a five-point Likert-type scale (ranging from 1 = “strongly disagree” to 5 = “strongly agree”).

A version derived from Kankanhalli et al. (2005), Lin (2007) was used to measure expected reciprocity (5 items.) The measure for intention to co-create OER (4 items) was adapted from a measure developed by Lin H. F. (2007). Measures for perceived effort (5 items) and increase in knowledge self-efficacy (4 items) were adapted from a measure developed by Kankanhalli et al. (2005). The measure for increase in Internet self-efficacy (3 items) was taken from a measure developed by Akhter (2014). The measure for increase in creative self-efficacy (3 items) was taken from a measure developed by Tierney and Farmer (2002). The measure for perceived privacy risk (4 items) was adapted from a measure developed by Hajli and Lin (2016), and the measure for perceived intellectual property risk (4 items) was developed from a construct developed by Lazarenko (2019). All items and scales can be found in Appendix A.

To enhance the clarity and readability of the translated measures, an educational technology expert with over 20 years of teaching experience in the United States and China was invited to make an independent bilingual assessment (Harkness and Schoua-Glusberg, 1998) of the complete translation.

### Data Collection

Responses were collected voluntarily and anonymously *via* a link from an online questionnaire survey platform, which required approximately 5–8 min to complete. Data were imported directly from the platform into SPSS 21.0 for analysis.

## Results

### Reliability and Validity of the Instruments

A total of 8 factors and 32 items were analyzed for confirmatory factor analysis (CFA). As shown in Table 1, the Cronbach’s alpha values for all factors were greater than the significance criterion of 0.7 (Hair et al., 2009), indicating that the adaptive measurement also had satisfactory reliability. Furthermore, AVE values were all greater than 0.5 (Segars, 1997), and CR values were all higher than 0.7 (Nunnally and Bernstein, 1994), demonstrating good aggregation validity.

**TABLE 1:** Reliability and validity analysis.

Factor	α	CR	AVE
Intention to co-create OER	0.92	0.92	0.75
Expected reciprocity	0.91	0.91	0.68
Increase in knowledge self-efficacy	0.86	0.86	0.60
Increase in Internet self-efficacy	0.89	0.90	0.75
Increase in creative self-efficacy	0.91	0.91	0.77
Perceived effort	0.85	0.85	0.53
Perceived privacy risk	0.71	0.78	0.52
Perceived intellectual property risk	0.71	0.78	0.58

### Overview of the Survey

Table 2 provides an overview of the participants’ mean scores and standard deviations on the survey. The results showed responses that ranged from 2.10 to 3.91. From the perspective of benefits, the factors that participants felt would affect their intention to co-create OER were, in descending order: increase in creative self-efficacy, expected reciprocity, increase in knowledge self-efficacy, and increase in Internet self-efficacy. From the perspective of cost, the factors that participants felt would affect their intention to co-create OER were, in descending order: perceived privacy risk, perceived intellectual property risk, and perceived effort.

**TABLE 2:** Descriptive statistics.

Factor		M	SD
Dependent variable	Intention to co-create OER	3.60	0.71
Independent variable (Benefits)	Increase in creative self-efficacy	3.91	0.68
	Expected reciprocity	3.86	0.70
	Increase in knowledge self-efficacy	3.86	0.64
	Increase in Internet self-efficacy	2.10	0.70
Independent variable (Cost)	Perceived privacy risk	3.45	0.68
	Perceived intellectual property risk	3.26	0.62
	Perceived effort	2.61	0.73

### The Effect of Determinants on Intention to Co-create Open Educational Resources

Regression analysis was conducted to investigate the relationships between intention to co-create OER and cost-benefit factors. As shown in Table 3, five out of seven factors from independent variables are predictors that collectively explain 46% (*R*^2^) of the variability in intention to co-create OER.

**TABLE 3:** Results of multiple regression analysis.

Dependent variable	Independent variable		*B*	SE	β	*t*	*R* ^2^
Intention to co-create OER	Benefit	Expected reciprocity	0.16	0.06	0.16	2.49*	0.46
		Increase in knowledge self-efficacy	0.19	0.09	0.17	2.09*	
		Increase in Internet self-efficacy	−0.01	0.08	−0.01	−0.16	
		Increase in creative self-efficacy	0.36	0.09	0.35	4.05**	
	Cost	Perceived effort	−0.09	0.04	−0.09	−2.11**	
		Perceived privacy risk	−0.14	0.05	−0.14	−2.68**	
		Perceived intellectual property risk	0.07	0.06	0.06	1.21	

*p < 0.05; **p < 0.01.

From the perspective of benefits, expected reciprocity (*t* = 2.49, *p* < 0.05), increase in knowledge self-efficacy (*t* = 2.09, *p* < 0.05), and increase in creative self-efficacy (*t* = 4.05, *p* < 0.05) were significant factors that positively predict the intention to co-create OER, while increase in Internet self-efficacy was found to not have a significant correlation. Therefore, H1, H2, and H4 were confirmed, while H3 was rejected.

From the cost perspective, perceived effort (*t* = −2.11, *p* < 0.05) and perceived privacy risk (*t* = −2.68, *p* < 0.05) were significant factors that negatively predict the intention to co-create OER, while perceived intellectual property risk was found to not have a significant correlation. Therefore, H5 and H6 were confirmed, while H7 was rejected.

## Discussion and Conclusion

### Discussion

Behavior intention is a critical, unbiased predictor of actual behavior. Understanding the antecedents of intentions increases our understanding of the intended behavior (Krueger et al., 2000). This study explored factors that influence the intention to co-create OER from a cost-benefit perspective as described by SET.

In this study, expected reciprocity, the core element of SET (Cropanzano and Mitchell, 2005), proved to have a significant positive influence on the intention to co-create OER. Students expected that when they co-created knowledge for OER repositories, they would get knowledge in return or get answer or respond when they are in need. In addition, they also value the opportunity to expand the scope of their associations with the online world. These direct benefits motivate learners to be more willing to engage in knowledge contribution behavior (Wasko and Faraj, 2005).

Contrary to our prediction, the increase of Internet self-efficacy was not an important factor motivating them to participate in the co-creation of OER. This might be explained by the age range of the participants. The participants who took part in the survey were between the ages of 18 and 22. They are part of a generation that has grown up with the Internet (Kolikant, 2010) and integrated it into almost every aspect of their everyday lives (Helsper and Eynon, 2010); for this reason they have been described as digital natives (Prensky, 2001). Therefore, increasing their Internet self-efficacy through the co-creation of OER may not be their primary concern.

Instead, consistent with our hypothesis, students value the increases in creative self-efficacy and knowledge self-efficacy that come with the co-creation of knowledge in OER repositories. Creative self-efficacy has demonstrated associations with creativity among individuals (Tierney and Farmer, 2004) as well as work teams (Richter et al., 2012). Creativity is a key modern-day skill (Voogt and Roblin, 2012) for life in the networked information society of the 21st Century. However, creativity is difficult to achieve (Mann, 2006) through college courses alone. Many studies have shown that interaction and cooperation in a virtual learning community can be a vital way to develop creativity in an academic context (Faraj et al., 2011; Greenhow et al., 2011). Furthermore, a relationship between creative self-efficacy or creativity and OER communities has been suggested by a small number of studies (Tosato and Bodi, 2011). Generally, the findings from this study extend those of previous research.

As mentioned above, this study showed that the increase in knowledge self-efficacy is a significant motivator for the intention to co-create OER. People increase their knowledge self-efficacy when they share useful information with others (Lu and Hsiao, 2007). Knowledge is recognized as a most important economic resource (Penrose, 1959) in the knowledge economy era. More and more undergraduates are aware of the limitations of traditional face-to-face universities in knowledge dissemination. They are more eager to broaden the scope of their education through blended learning (Bonk and Graham, 2006; Graham, 2006). As the most large-scale open source movement, OER support quality education that can ultimately lead to the realization of inclusive knowledge societies (UNESCO, 2017). Therefore, it is easy to understand why students may view the increase of knowledge self-efficacy as a core intrinsic benefit of OER co-creation.

Contrary to what we predicted, perceived intellectual property risk has no significant influence on the intention to co-create OER. The balance between openness and ownership over intellectual property is an ever-present issue in OER initiatives (Atkins et al., 2007). In fact, students’ average response value for perceived intellectual property risk (*M* = 3.26, SD = 0.62) was much higher, reflecting the widespread concerns about intellectual property rights in the context of OER. To explain the non-influence of intellectual property issues, we propose that students are aware of intellectual property risks, but this does not affect their intention to contribute knowledge. This is because most of the knowledge that undergraduates believe they could contribute is common knowledge instead of professional, domain-specific, or innovative knowledge. A common worry is that sharing or contributing these more valuable forms of knowledge will reduce one’s power, influence, and authority (Cabrera and Cabrera, 2002; Ipe, 2003), but these concerns do not apply to common knowledge.

Students’ average response value for perceived effort (*M* = 2.61, SD = 0.73) is the next-to-lowest in the survey, which indicates that codifying and clarifying the knowledge in OER repositories is no longer considered laborious by today’s undergraduates who possess high information literacy (Correa, 2016) and level of content knowledge (Bryan and Clegg, 2019). However, under normal conditions it is desirable to produce more output with less input, because this leads to higher efficiencies (Duran et al., 2016). Therefore, there is still a significant negative relationship between expected effort and the intention to co-create OER.

Consistent with our hypothesis, perceived privacy risk is negatively related to the intention to co-create OER. People are concerned that online platforms will capture or collect too much of their personal information (Hajli and Lin, 2016). They are also worried that unknown third parties will access their personal information (Punj, 2017). The findings from this study confirmed these points in the context of OER.

### Contributions and Implications

At the most basic, theoretical level, this study provides a framework from the perspective of SET to analyze the cost-benefit factors that influence the intention to co-create in the context of OER, while previous studies have focused more on knowledge sharing (Bock and Kim, 2002) or value co-creation in public service (Füller, 2010; Preikschas et al., 2017). Furthermore, previous studies have yielded contradictory results on the relationship between expected reciprocity and knowledge sharing (Endres and Chowdhury, 2013). The findings of this study add to the evidence for a significant positive relationship between expected reciprocity and co-creation of OER in the context of OER. Additionally, intellectual property concerns have long been regarded to be the most important factor hindering the development of OER (Joyce, 2007). However, this study has shown that, for certain groups of people, the awareness of copyright risks does not have a negative impact on their intention to co-create; this result is of great significance for the future development of OER.

Three practical strategies are recommended to promote the intention to co-create OER, based on the findings of this study. First, reciprocity is a behavioral indicator for the emergence of a new community (Aviv and Ravid, 2005), and indeed one of the defining attributes of any community (Wellman and Gulia, 1999), while the norm of reciprocity helps guarantee a high level of communication (Casaló et al., 2013). Therefore, to encourage reciprocity behavior, OER stakeholders should form more and more OER communities and develop reciprocity norms, either deliberately or spontaneously, according to the subject matter or the participants’ occupations or hobbies. Second, knowledge and use of Creative Commons copyright licenses should be popularized and promoted among undergraduates to encourage them to correctly re-use, re-mix, and distribute OER. Creative Commons licenses are a suite of copyright-based licenses that define terms for the distribution and re-use of creative works (Hartley, 2005) in a way that creates a balance inside the traditional “all rights reserved” setting that copyright law creates (Creative Commons, 2017). It is vital to promote good copyright awareness and behavior that can provide a broad space for sharing or co-creating professional knowledge in the future. Finally, easy-to-use online resource editing tools with common formats should be provided for use with OER repositories; this can effectively reduce users’ codification effort and improve co-creation efficiency. There are already some repositories that are increasingly focused on the development of online codification tools. For example, OER Commons provides the tool of Open AUTHOR to better support OER co-creation.

### Limitations and Directions for Future Research

The main limitation of this study is the use of a homogeneous sample of individuals with similar ages, cultural backgrounds, and MOOC experience. Future research should, therefore, explore other ages, cultural backgrounds, and types of online learning experience to help broaden the universality of the results. Additionally, some studies have shown that there is an inverted U-curve between expected reciprocity and behavior intention or actual behavior (Fyrand, 2010). This phenomenon should be specifically explored using reciprocity theory in the context of OER co-creation. Furthermore, the relationship between the intention to co-create OER and actual co-creation behavior is also worth exploring.

## Data Availability Statement

The raw data supporting the conclusions of this article will be made available by the authors, without undue reservation.

## Author Contributions

XW: writing—original draft preparation and formal analysis. RH: formal analysis. HY: conceptualization and methodology. All authors contributed to the article and approved the submitted version.

## Conflict of Interest

The authors declare that the research was conducted in the absence of any commercial or financial relationships that could be construed as a potential conflict of interest.

## Publisher’s Note

All claims expressed in this article are solely those of the authors and do not necessarily represent those of their affiliated organizations, or those of the publisher, the editors and the reviewers. Any product that may be evaluated in this article, or claim that may be made by its manufacturer, is not guaranteed or endorsed by the publisher.
